# Tourism as a Tool in Nature-Based Mental Health: Progress and Prospects Post-Pandemic

**DOI:** 10.3390/ijerph192013112

**Published:** 2022-10-12

**Authors:** Ralf C. Buckley, Mary-Ann Cooper

**Affiliations:** 1 School of Environment & Sciences, Griffith University, Southport, QLD 4215, Australia; 2 Instituto Profesional de la Fundacion Duoc UC de la Pontificia, Universidad Católica, Viña del Mar 2336, Chile

**Keywords:** parks, wellbeing, tour guide, psychology, economics, COVID-19

## Abstract

The healthcare sector recognises the role of nature in mental health. The tourism sector is equipped to take people to national parks. The conservation sector gains support from visitors. Theoretical frameworks for mental health benefits from nature tourism include: tourism destinations and activities; tourist personalities and life histories; sensory and emotional components of tourist experiences; and intensity and duration of memories. Mental health deteriorated worldwide during the COVID-19 pandemic. Recovery of global economic productivity requires immediate, accessible, affordable mental health measures at national scales, and nature-based approaches provide the best option. Different countries have adopted a variety of public, private, or voluntary mechanisms. Some focus on design of activities, others on provision of facilities. Costs and implementation depend on key research questions: marginal benefits of nature tour guides or psychologists compared to self-guided nature experiences; comparisons between repeated brief visits and one-off nature holidays; effects of biodiversity, flagship species, and scenic or wilderness quality; and differences between individuals, depending on personalities, life histories, and mental health status and symptoms.

## 1. Introduction

Tourism can contribute to tourist wellbeing; and wellbeing has social and economic value, underpinning healthcare and health insurance. In particular, tourism takes people to parks and nature destinations, where contact with nature can improve their mental health. Here, we compare and analyse what practical methods and measures have been trialled, in different countries, to take advantage of the overlap between nature tourism and nature therapy, and with what outcomes. We review progress and prospects, in both theory and practice, at four scales: global, national, sectoral, and individual. We consider the role of nature tourism in post-pandemic recovery of population-scale mental health, as a key component in workplace economic productivity.

We propose that there is very considerable potential, and profitable opportunities, for the tourism sector to contribute to nature-based mental healthcare. We argue that approaches taken to date by various countries have been rather ineffective or small scale. Larger-scale approaches are now under adoption, but several key research questions remain unanswered. Answers to these questions are important for all three of the sectors involved, namely tourism, healthcare, and conservation. They are also important for national governments to be able to adopt nature-based approaches to mental healthcare as an immediate, accessible, and affordable mainstream measure in post-pandemic economic recovery.

## 2. Theoretical Frameworks

Theoretical frameworks in this field are still under development, and there is as yet no generally accepted consensus approach. At broad scale, there have been multiple parallel lines of research on tourism and wellbeing, focussing on different aspects and subsectors (Table 1).

**Figure 1:**
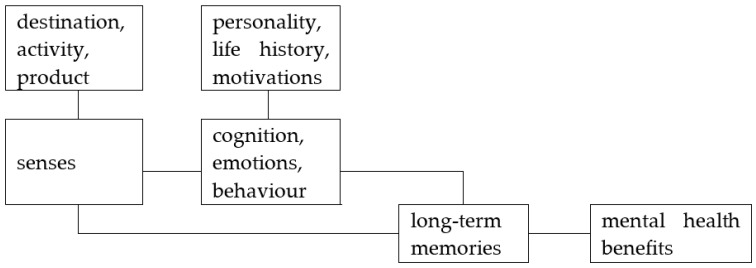
Current Theoretical Framework for Tourism Mental Health Benefits.

Recently, links between tourism and mental health have been explored through a series of sequential approaches, each with slightly different theoretical frameworks (Table 2). These focus on mental health as a component of clinical medicine and public health, rather than lifestyle health and wellbeing. The most recent and general of these frameworks includes: tourism destinations and activities; tourist personalities and life histories; sensory and emotional components of tourist experiences; and intensity and duration of memories (Figure 1). The main components of this framework have a long history of research as independent topics, with recent research on senses [23,24,25,26,27,28,29,30], emotions [31,32,33,34,35], and memories [36]. Mechanisms derived from recent research on tourism and mental health are closely analogous to those developed during earlier research on tourism experience value [37,38].

The focus on mental health rather than wellbeing has been pursued particularly by an Australian research group, using an argument that is essentially economic. Within the healthcare sector, wellbeing is a term used largely in reference to discretionary, patient-funded lifestyle medicine. This is treated as marginal to mainstream clinical and public healthcare, funded through medical insurance and government budget allocations. Effects of tourism on wellbeing are seen as personal benefits, paid for and received by individual tourists. Improvements in mental health, in contrast, are seen as contributing to broader society and economy. Governments routinely maintain economic statistics and analyses on costs of mental health [39,40]. The research requirements for measuring effects of tourism on mental health, however, are set by the expectations of medical rather than business research [41]. This approach has shown that parks worldwide have an economic value via visitor mental health of USD 6 trillion per annum, including USD 2.1 trillion from reduced healthcare costs and improved workplace productivity [42]. Mental health approaches have now been expanded across tourism and recreation research more broadly [43,44].

**Table 2:** Recent Development of Frameworks for Mental Health of Nature-Based Tourists.

Year	Approach	Framework	Refs
2016	Tourism mental health outcomes	Framework for mental health gains from outdoor parks and nature tourism	[45]
2019	Tourism options within healthcare	Commercial nature tourism can provide components missing from health sector	[46]
2019	Health services value of parks	Parks tourism boosts visitor mental health, value USD 6 trillion pa worldwide	[47]
2020	Mental health tourism model	Qualitative analysis of mental health gains from nature tourism charity challenge	[48,49]
2020	Causal direction parks and health	Causality: visiting parks increases happiness, rather than the reverse	[50]
2021	Nature, COVID, and mental health	Mental health depressed by nature deprivation during COVID lockdowns	[51]
2021	Parks and post-COVID recovery	Economic recovery post-pandemic needs nature-based tourism	[52,53]
2021	Mental health and parks infrastructure	Including mental health outcomes changes best parks tourism infrastructure options	[54]
2021	Destination image and marketing	Nature tourism destinations and enterprises market mental health	[55]
2021	HSV as important ecosystem service	Comprehensive models of ecosystem services should include mental health services value, HSV	[56]
2022	Framework tourism and mental health	Tourism and mental health, review and framework for future research, focus on nature-based tourism	[57]
2022	Sense-emotion- memory model	Sensory and emotional experiences and memories, wildlife tourism	[58]
2022	Productivity value of parks tourism	Economic productivity boost and health cost savings from park tourism	[42]
2022	Human capital value of tourism	Wildlife tourism generates economic counterflow via human capital value	[59]

The frameworks outlined in Table 1 and Table 2 and Figure 1 are tourism research frameworks. Different theoretical frameworks are used within healthcare and conservation research, as outlined in Table 3, because the sectors have very different aims and structures.

In the healthcare sector, the benefits of nature for mental health are well established and accepted at proof-of-concept level; but they are not yet converted to the details of dose, response, and duration in relation to patient symptoms and personality, required to construct prescriptible courses of psychotherapy [41,60,61,62,63,64,65,66,67]. Evidence to date indicates that a minimum of 2 hr/wk nature contact is required to achieve any therapeutic effect [66]. To achieve adherence to therapy and sustained behavioural change seems to require a larger dose, e.g., 4 hr/wk, and a minimum course duration of 12 weeks [45,48]. These figures are likely to differ between: patients and symptoms; place and intensity of outdoor activities; and whether they are led by a qualified psychologist or nature guide, or unguided. So-called green prescriptions, as currently implemented, seem to be much too limited to be effective, and do not include any practical means of implementation [57].

From a conservation perspective, there are two central considerations. Conservation policies, and declaration of conservation reserves, need political support, and this is derived partly through the economics of ecosystem services. Practical management of protected areas needs cash, either from government budget allocations, or other sources, or both. These can differ considerably between countries, and between conservation areas in the same country. Visitor mental health is a newly recognized mechanism to calculate one significant economic value of nature and national parks [42,47]. It could also be harnessed to provide direct cashflow to parks agencies, but at the risk of increased ecological impacts and loss of control.

## 3. Scales and Social Context: Trends and Patterns

### 3.1. Global Scale: Access to Natural Resources and Ecosystem Services

Mental health benefits for parks and nature tourists are a form of cultural ecosystem service [56]. At global scale, human access to natural resources, including ecosystem services, follows four main models, either in series or in parallel (Table 4).

The same resource or service may be available via each of these models, at different times and places; or even at the same time and place, as is often the case for drinking water. Mental health benefits from nature depend on access to relatively undisturbed natural areas, either public, communal or private. Currently, most of the world’s population is urbanized, and access either to private rural lands, urban greenspace, or public lands such as national parks, is quite inequitable. In developed nations, nature-based mental health is available largely at level 3 above, taxpayer-funded public lands; but increasingly, it is moving to level 4, as commercial enterprises seek to capitalise on increasing demand and limited supply.

At present, private estates, and private or rented nature holiday homes, are available only to wealthier individuals. Many rural residents have access to rural production lands, such as farmland. Public urban greenspace is unevenly distributed, with higher house prices correlated with greater greenspace. At least a third of urban populations in developed nations, and two thirds in newly industrialized nations, do not visit national parks [47,68]. Reasons may include: lack of time or money; social and cultural preferences; or individual life histories. There are also cultural differences in preferred activities within national parks: not everyone visits principally for nature appreciation.

At level 4 in Table 4, there is competition between private stakeholders, for access to natural resources or ecosystem services. Enterprises can profit by: capturing exclusive use or access rights to natural areas with high environmental quality; obtaining preferential government funding to provide a public service; constructing and marketing retail products directly to consumers of nature-based mental health; or a combination of the above. Nature tourism enterprises whose business models rely on access to public protected areas, find themselves in political as well as market competition, depending on the types of access rights they hold. For example, those who have a fixed-site private foothold inside public protected areas, via various mechanisms, seek to exclude those who rely on mobile use of public infrastructure [69]. Demand for access is increasing, as tourism enterprises use mental health to enhance their marketing. In future, private healthcare and health insurance corporations, much larger and more powerful than the tourism sector, may buy tourism enterprises simply to acquire their parks access rights. This already happens through acquisitions within the tourism sector.

### 3.2. National Scale: Approaches to Nature-Based Mental Healthcare

Different countries have taken a range of different approaches to incorporating nature-based components in their national mainstream mental healthcare systems. Some of these include or rely on commercial tourism models; some on community outdoor recreation models; and some on public or clinical healthcare models. To date, tourism models have proved more successful, healthcare models less so. The option of combining the two sectors has rarely been adopted, though the most recent measures within China, outlined below, appear to be moving in that direction.

Some countries simply have strong cultural incentives for individual outdoor recreation, and social mechanisms that provide easy access to natural areas. In New Zealand, backcountry hiking is known as tramping, and is a powerful component of the national psyche. There is an extensive system of national parks, and most people have access to them. In Norway, Sweden and Finland, there is a long and continuing tradition of outdoor recreation, termed *friluftsliv*, fresh-air life; and public hiking access to private as well as public lands, through the historical doctrine of *allemansrett*.

Historically, much of the UK was accessible via public walking or horseback rights-of-way, though these are now greatly reduced through long campaigns by private landholders. In countries such as USA, Canada, Australia, and Chile, there is no public access to private lands. There are national park systems with maintained hiking tracks and trails. There are traditions of outdoor recreation, but these apply only to some population subsectors. They also include motorised and consumptive activities, though these are declining in popularity nationally, relative to lower-impact activities such as hiking, kayaking, and birdwatching.

The key issue from a mental health perspective in these countries, however, is that there are substantial population sectors who do not take part in any of these outdoor activities, or visit national parks or other public lands at all: either independently, or as clients of tourism operators or outfitters, as nature and adventure enterprises are known in North America. From a public health perspective, therefore, one of the critical considerations is, how to encourage and enable park visits by nature-deprived population subsectors. There has only been limited research to date on the socioeconomic and demographic characteristics of those subsectors [68].

In both USA and UK, there have been rather half-hearted and unsuccessful attempts to link outdoor recreation activities into mainstream healthcare funding. In the USA, healthcare funding relies heavily on employer-funded private health insurance. The federal government established a funding program aimed at using exercise to reduce diabetes, which reimbursed health insurers for the costs of private employees enrolling in provider programs. Insurance brokers took advantage of this to promote outdoor exercise programs to large health insurers, using patented software intended to match patients to providers, with automated tracking and billing [57]. To increase the number of patients, they marketed this approach to employers and health insurers as a preventive nature-based mental health program, applicable to all staff, not only the smaller proportion with diabetes. This seems to have started in 2018. The brokers still exist, and so does the diabetes funding, but as of September 2022, the link to nature-based exercise for mental health seems to have disappeared.

In the UK, the taxpayer-funded National Health Service commenced a large-scale approach under the title of Social Determinants of Health, SDH. One component of this was intended to improve equitable access to outdoor nature-based exercise opportunities. The actual measures taken, however, were rather ineffective. The national government intended to fund 100 positions within local governments: individuals who would act as information brokers between doctors and local outdoor voluntary recreation groups, such as hiking clubs or competitive mountain-running associations [57]. The therapeutic role of wetland visitor centres has also been mentioned [70]. It is not clear how many positions were actually funded, nor how many patients were referred to voluntary groups, nor whether those groups were able to accept new members in poor mental health. The SDH program still exists [71], but no longer mentions nature-based mental health.

In Australia, healthcare is funded through a combination of: individual-premium private health insurance; budget allocations to state government health portfolios; and direct patient payments. Mental healthcare is substantially underfunded by government, and relies on semi-voluntary organisations that receive both government funds and donations. There are national educational marketing programs aiming to overcome historical stigma in discussion of poor mental health. There are private social-purpose enterprises that operate participant-funded nature-based mental health programs, using charity-challenge tourism business models [57]. The largest of these [48] is marketed principally to urban mothers, and has successfully attracted 1% of the national adult female population to date. The fundraising component, >$40 million to date, was historically donated to mental health organisations, but that has now changed to a cardiac health organisation. The principal mental health contribution is for the participants themselves, and this remains effective. The main function of the fundraising component is to prevent participants dropping out of the program, which runs for 12 weeks. There are also enterprises offering a broader range of shorter small-group programs with a stronger tourism focus, where the charity challenge fundraising is parallel to the activity component. These enterprises survived the COVID-19 disruption, and remain operational.

In Japan, there is a long tradition of peaceful contemplation of natural landscapes, especially those with forests and streams. In recent decades, this has been formalised as *shinrin-yoku*, forest bathing, which has also been exported worldwide as a lifestyle medicine concept. There are forest therapy societies and associations, and a government-run certification program for forest therapy destinations. There do not seem to be any published data, at least in English, as to what proportions of their populations take part in *shinrin-yoku* programs. Some forest national parks, but not all, are accessible via public transport from metropolitan cities. There are similar though less well-known programs in Korea.

In China, there is a new and rapidly expanding government program, apparently the only national-scale, cross-portfolio attempt at nature-based mental health therapy [72,73]. Initiated by the national forestry agency under the name of Forest Therapy Hubs, it has now been brought into a joint initiative by national health, tourism, and land management agencies, under a title which translates as Healthy Life Bases, effectively nature therapy centres. These are visitor centres designed for self-guided mental health therapy, e.g., via short interpretive forest trails. Details differ between sites, and it seems that they combine redesign and rebranding of existing visitor centres, and newly constructed facilities. The goal is to construct 1200 of these bases nationwide. The program started last year, and ~100 had been opened by the end of the year [73]. Improvements in public-transport access, and options for on-site guiding and counselling, are apparently under consideration but not yet operational.

### 3.3. Sectoral Scale: Patterns and Updates

Nature and adventure tourism, international as well as domestic, are substantial and profitable subsectors of the tourism industry. There are powerful and long-running campaigns by tourism industry advocates in a number of countries, to extend private commercial tourism access to public protected areas and other public lands. Until recently, these did not include mental health components. Similarly, research on tourism and wellbeing has only recently focussed on nature-based products. Currently, there seem to be two relevant trends within the tourism sector. The first is to include mental health benefits in marketing for nature tourism products and destinations, more explicitly than in the past [55]. That applies for both fixed-site accommodation and facilities, and mobile tours. The second is the expansion of charity-challenge events and programs marketed specifically for participant mental health, such as those outlined above.

To date, however [46], we have not seen tourism products restructured and rebranded directly as mainstream nature therapy products. This seems to be a substantial opportunity. In some countries, private patient-funded psychotherapies are very widespread and commonplace amongst urban populations, and the therapists concerned have considerable latitude in how they construct their products. It seems that they could quite easily partner with outdoor tour guides and nature tourism enterprises, to offer nature-based psychotherapies at upper-tier prices. That may not do much for public mental health more widely, but it would provide profitable new commercial opportunities.

More broadly, parks and nature contribute to the mental health of independent tourists, with corresponding economic benefits [47,59]; but in general, national public and clinical healthcare systems have not yet taken advantage of this. In Australia, for example, the national government healthcare program, known as Medicare^®^, has a funding code for 10 sequential 1 h sessions of small-group counselling, by a single psychologist, for 6–10 patients simultaneously. That option, however, requires diagnosis and prescription by general medical practitioners, and seems to be little used. Some psychologists offer counselling outdoors, but sessions are only 20 min, well below the 2 h/wk effectiveness threshold.

Therefore, it appears that a considerable public mental health benefit could be achieved by relatively minor modification to the Medicare^®^ code definitions, with partnerships between psychologists and nature tour guides and enterprises to provide parks access permits, safety and logistics, and nature interpretation. This option would not require rebranding or medical certification of tourism products as therapies. It would require some additional public funding for the extended Medicare^®^ service. It would also require funding, either public or private depending on patient means, to cover the costs of the tourism components. Given the very large scale of pandemic-related mental health deterioration, however [74,75], these costs would represent a small investment with a large return.

From a healthcare perspective, key considerations are the design of courses of treatment, relative to patient symptoms and characteristics. Evidence to date has converged on courses with at least 2 and preferably 4 hr/wk, for at least 12 weeks. This is 2–4 times the maximum length of individual treatments, and double the overall duration, of current standard courses for publicly funded psychotherapy and physiotherapy. It is less, however, than some private psychotherapies, so it is within the envelope of current mental health treatment systems.

We do not yet have evidence as to whether a single intensive nature holiday may be more or less effective, from a mental health perspective, than a 12-week course of brief weekly nature activities. Medical funding and insurance systems can accommodate both one-off major operations, and extended courses of treatment, so either option should be feasible. There is also no medical evidence as yet, as to whether a skilled nature tour guide boosts mental health outcomes from time in nature; or only knowledge, enjoyment and satisfaction. Nor is there any medical evidence whether being accompanied by a psychologist adds anything to mental health benefits from direct experience of nature itself. Finally, we do not yet know how long mental health benefits of nature experiences may last. To date, we only have evidence that: mental health benefits improve with pristineness and biodiversity [19,76,77,78,79,80,81]; intense individual experiences remain memorable for many decades [58]; and fade-out in wellbeing after non-nature vacations can extend over months or years [10]. Additional evidence on each of these topics is therefore required, in order to refine nature therapy designs.

Nature therapies may also need to be modified to suit different patients. Physical capabilities may differ greatly depending on age, and on factors such as fitness and body mass index; and mental health gains from nature may depend strongly on personality factors such as nature relatedness [82] or connectedness [83,84,85], and on life history factors such as ageing [22,86], and childhood exposure to the outdoors [27,47,83,87,88,89,90]. Different individuals may need different incentives or social levers to start and continue nature therapy courses, depending whether any barriers are individual, cultural, social, or geographical and economic [45].

From the perspective of the nature conservation sector, there is a distinction between political support for improved government budgets, and immediate financial support by charging for access or activities, either for individual park visitors, or via permitting processes for commercial enterprises. Currently, commercial tourism enterprises in many countries operate in public as well as private lands. Protected areas already have publicly funded access, infrastructure, and visitor facilities, as well as the primary nature attractions. These represent a public subsidy, along with the taxpayer-funded costs of managing ecological impacts. Fees differ greatly, depending on types of activity and local politics and regulations.

In some countries, notably developing nations in African wildlife tourism destinations, conservation funding relies heavily on tourism, and at least some commercial tourism enterprises make net positive contributions to conservation [59,91], through up-market, minimum-impact lodges, many on private reserves or communally owned lands. In other countries, in contrast, including some developed nations, there are a growing number of commercial property developers who have obtained permits to construct permanent lodges inside public national parks, with net negative effects on ecology, social equity, and regional economies [69]. To date, the healthcare sector has not copied these approaches. If tourism and healthcare enterprises form partnerships, however, as we currently predict, then their political clout and potential adverse impacts will greatly exceed that of tourism alone.

### 3.4. Individual Scale: Psychological Mechanisms

At the most fine-grained level, the therapeutic effects of nature-based tourism differ between individuals. Individual differences are well established in psychological research, and tour guides and psychologists both learn to customise their approaches to their clients’ interests, personalities, and life histories. In tourism mental health and tourism experience value (Figure 1), different individuals may experience different senses and emotions during the same activity at the same place and time. This has been recorded in practice, e.g., for wildlife tourism [58] and adventure tourism [92]. Attitudes, motivations and memories differ correspondingly, with consequent effects of mental health outcomes.

Across the three sectors considered here, namely tourism, healthcare, and conservation, it seems to be tourism that has achieved the greatest practical recognition of these individual differences, which are central to targeted marketing, choreography by tour guides, and experience value for tourists. Learned tacit skills of nature tour guides could be applied to maximise the mental health benefits of outdoor tourism. From a theoretical perspective, psychological mechanisms for tourism experience value and tourism mental health are closely congruent [38,57]. As the healthcare sector takes greater advantage of nature-based therapies, the psychological skills of nature tour guides will achieve greater recognition.

## 4. Discussion: Research Priorities

In tourism research, there seem to be two priority topics. The first is to measure tourist wellbeing outcomes using mental-health methods and terminology, so that the therapeutic benefits of tourism products can be compared to those of courses designed directly as therapies. Currently, mental health benefits may be marketed as one additional reason to purchase a particular tourism product or visit a particular tourist destination, but without evidence that would be accepted in the healthcare sector.

The second is to analyse the psychological drivers and factors, at individual scale, that determine: what mental gains are achieved; by what mechanisms; and how long they last. Some people are happily challenged to climb a mountain in bad weather, and unhappily bored lying in the sun with a drink; whereas for others, the reverse applies. Some outdoor tourists want active adventure thrills, even if they involve risks and fear. Others want more contemplative nature experiences, such as scenery, waterfalls, wildlife, or birdwatching. Currently, the tourism approach is simply to offer products of different types, and leave purchasers to choose. As digital tourism marketing becomes more tightly targeted to individual consumers, however [93], an understanding of mechanisms will gain increased commercial significance. Currently, tourism research does consider satisfaction and future motivations, and fade-out of self-perceived wellbeing post-vacation; but it does not yet track in detail how satisfaction from one tourist experience may gradually be converted, post-vacation, to motivation for future experiences.

Within the healthcare sector, the priority is to design, construct, test and implement nature-based mental-health therapies within mainstream healthcare systems, funded by health insurers and government health portfolios as well as individuals. This will include systems for diagnosis and customisation to individuals, using the terminology of patients rather than clients. The measure of value is via long-term mental health outcomes, rather than short-term customer satisfaction. The logical approach is for the healthcare sector to take advantage of accrued expertise within tourism, to provide all the outdoor components that are unfamiliar within healthcare. This has not yet happened, but healthcare research could focus on testing the mental health of nature tourism products that already exist, relative to the psychological characteristics of the tour clients.

For conservation, there would seem to be three immediate research priorities. The first is in the economics of health services value, the mental health value of visiting parks, at national or state government scale. Currently, there are calculations showing how visits to national parks increase economic productivity and reduce healthcare costs [42]. As yet, however, there has been no attempt to calculate marginal returns, via these mechanisms, on increased investment in the budgets of protected area management agencies. The second is in the practical politics of using parks for nature-based mental health therapies. On the one hand, partnerships between health insurers and outdoor tourism enterprises may provide financial opportunities for both; but on the other, both may arise at the expense of conservation and public parks agencies [69]. Therefore, those agencies would be wise to devise and test appropriate access control and fee systems immediately, before they are taken unawares. The third priority is to test the differential mental health effects of different components of conservation, such as biodiversity and flagship species, and how these differentiate national parks from urban greenspace.

## 5. Conclusions: Progress and Prospects Post-Pandemic

There has been considerable recent progress in research on nature tourism and mental health. Economics approaches have shown that the value of parks and nature tourism for human capital and mental health is very substantial, large enough to merit more detailed research on psychological mechanisms. Previous research on individual personalities, tourism settings and activities, sensory and emotional experiences, and memories and wellbeing, have been integrated to construct a general mechanistic framework, which can provide a basis for finer-grained quantification in future research. The principal tourism research priority is to quantify the types, intensities, and durations of therapeutic outcomes from a variety of different tourism products, in relation to: tourist or patient personalities and life histories; tourist setting and activity; guiding and/or counselling; and specific components of scenery, vegetation, or wildlife, such as biodiversity or flagship species, and specific sensory experiences.

Commercial and policy opportunities and risks have been identified in the tourism, healthcare, and nature conservation sectors; and how these play out will depend on differences between countries. At present, the most detailed design to be implemented and tested in practice at large scale, is an activity-oriented approach, a 12-week program of energetic weekly small-group national-park hikes, with a set of social levers to encourage high participation rates and low drop-out. This, however, may not be suitable for everyone. An alternative approach, under large-scale construction and testing in China, is through a large number of fixed-site self-guiding nature therapy facilities, in public parks and forests nationwide. Not yet trialled, but worthy of research, would be a combination of the activity and facility approaches, with multiple repeat visits and activities at or adjacent to readily accessible parks visitor centres.

All of these considerations, already important worldwide per-pandemic, have become increasingly urgent and significant post-pandemic. There is now very extensive research detailing the effects of COVID-19 itself, and associated personal and social disruptions, on: deterioration in mental health [51,74,75,94,95,96,97,98]; access to, and enjoyment of nature during the pandemic [99,100,101,102]; and the effects of nature on maintaining mental health [103,104,105,106,107]. There is also a growing body of statistical and modelling information on the effects of the pandemic in decreasing economic productivity at various scales [108,109,110,111,112]. Governments are now urgently seeking to establish immediate and affordable national-scale public health programs to restore mental health and hence national economic productivity. They do not have time or funds to train and employ three times the number of certified psychologists and psychiatrists, to match the tripling in frequency of poor mental health at peak pandemic.

What countries do already have, is national parks and nature. Individuals who were already accustomed to visit parks as part of their pre-pandemic lifestyles, took steps to continue even during lockdowns, and there have been surges in park visitation post-pandemic [113,114,115]. In both developed and newly industrialised nations, however, and urban areas in developing nations, there are substantial population sectors who do not visit parks, and may not have equitable access to urban greenspace. One constraint is opportunity, in time as well as money for access and transport [45,48]; but another is unfamiliarity, including cultural constraints and lack of childhood experience [47,68]. There are thus substantial sectors, one to two thirds of the population in many countries, whose mental health could benefit considerably from repeated, guided visits to existing national parks. From healthcare perspectives, that could combine 12-week, ~4 hr/wk small-group outdoor activities as in Australia, with fixed-facility Healthy Life Bases as in China. From a tourism perspective, it would combine outdoor nature, parks and adventure tourism enterprises and tour guides, with national parks destinations, visitor infrastructure, and interpretation centres and programs. The role of nature tourism in mental healthcare has thus become especially important and significant.

## Figures and Tables

**Table 1:** Threads and Terms in Tourism, Wellbeing and Related Research Topics.

Approach	Framework	Refs
Social tourism	Publicly funded tourism for economically disadvantaged, e.g., spas in Eastern Europe	[1]
Medical tourism	Travel for specific medical procedures, including cosmetic, e.g., to cut costs	[2]
Wellness tourism	Specialist subsector, spas, yoga, wellness retreats, lifestyle medicine	[3]
Wellbeing tourism	Wellbeing outcomes from all types of tourism, including both hedonic and eudaimonic wellbeing	[4,5,6,7,8]
Vacation duration	Duration of pre- and post-vacation wellbeing fade-in and fade-out of wellbeing effects	[9,10]
Arts + shops tourism	Wellbeing outcomes from arts, music, and shopping tourism in urban settings	[11,12,13]
Transformative	Transformative phenomenology of adventure tourism, outdoor sport and recreation	[14,15]
Vocational	Positive psychology, charity challenges	[16]
Restorational	Tourism as a stress recovery mechanism	[17,18,19,20,21]
Healthy ageing	Tourism can maintain health of elderly	[22]

**Table 3:** Frameworks for mental health in tourism, healthcare and conservation.

Sector and Framework Components
**Tourism**
***Subsectors:*** urban, rural, nature; indoor, outdoor; broad cf narrow
***Funding:*** individual tourists, within competitive commercial markets
***Focus:*** motivations and marketing, satisfaction and repeat bookings
***Products:*** destination, activity, guides, level of luxury
***Research:*** intensity of experiences, duration of memories, effects on perceived values
**Healthcare**
***Subsectors:*** clinical, public, lifestyle, traditional
***Funding:*** insurance, taxpayers, individual patients, combinations
***Focus:*** diagnosis, prescription, treatment, evaluation
***Products:*** prescriptible courses of nature-based psychotherapies
***Research:*** design of therapies, implementation and funding models
**Conservation**
***Subsectors:*** public protected areas, private conservation reserves, urban greenspace
***Funding:*** national or state governments, donors and private sources, local governments
***Focus:*** biological diversity and ecosystem services, public recreation opportunities
***Products:*** investments in visitor infrastructure, charges for visitor access and activities
***Research:*** mechanisms to improve political and financial support for conservation

**Table 4:** Systems for Human Access to Natural Resources & Ecosystem Services.

	Access System	Cost Mechanism for Individual User
1	individual open access	free except for cost of harvesting
2	communal control	cheap but competitive against other users
3	government provision	pay-per-connection or pay-per-use utility
4	commercially privatized	via wholesale and retail markets

## Data Availability

All data included in article.
